# Diagnostic Accuracy of Abbreviated Bi-Parametric MRI (a-bpMRI) for Prostate Cancer Detection and Screening: A Multi-Reader Study

**DOI:** 10.3390/diagnostics12020231

**Published:** 2022-01-18

**Authors:** Giorgio Brembilla, Francesco Giganti, Harbir Sidhu, Massimo Imbriaco, Sue Mallett, Armando Stabile, Alex Freeman, Hashim U. Ahmed, Caroline Moore, Mark Emberton, Shonit Punwani

**Affiliations:** 1 Centre for Medical Imaging, University College London, London W1W 7TS, UK; harbir.sidhu.13@ucl.ac.uk (H.S.); sue.mallett@ucl.ac.uk (S.M.); s.punwani@ucl.ac.uk (S.P.); 2 Department of Radiology, IRCCS San Raffaele Scientific Institute, Vita-Salute San Raffaele University, 20132 Milan, Italy; 3 Department of Radiology, University College London Hospital NHS Foundation Trust, London NW1 2PG, UK; f.giganti@ucl.ac.uk; 4 Division of Surgery & Interventional Science, University College London, London NW3 2PS, UK; caroline.moore@ucl.ac.uk (C.M.); m.emberton@ucl.ac.uk (M.E.); 5 Department of Advanced Biomedical Sciences, University Federico II, 80131 Naples, Italy; massimo.imbriaco@unina.it; 6 Department of Urology and Division of Experimental Oncology, Urological Research Institute, Vita-Salute San Raffaele University, IRCCS San Raffaele Scientific Institute, 20132 Milan, Italy; stabile.armando@hsr.it; 7 Department of Pathology, University College London Hospital NHS Foundation Trust, London NW1 2BU, UK; alex.freeman2@nhs.net; 8 Imperial Urology, Charing Cross Hospital, Imperial College Healthcare NHS Trust, London W6 8RF, UK; hashim.ahmed@imperial.ac.uk; 9 Imperial Prostate, Division of Surgery, Department of Surgery & Cancer, Faculty of Medicine, Imperial College London, London SW7 2BX, UK; 10 Department of Urology, University College London Hospitals NHS Foundation Trust, London NW1 2PG, UK

**Keywords:** magnetic resonance imaging, prostate cancer, biparametric MRI, screening

## Abstract

(1) Background: There is currently limited evidence on the diagnostic accuracy of abbreviated biparametric MRI (a-bpMRI) protocols for prostate cancer (PCa) detection and screening. In the present study, we aim to investigate the performance of a-bpMRI among multiple readers and its potential application to an imaging-based screening setting. (2) Methods: A total of 151 men who underwent 3T multiparametric MRI (mpMRI) of the prostate and transperineal template prostate mapping biopsies were retrospectively selected. Corresponding bpMRI (multiplanar T2WI, DWI, ADC maps) and a-bpMRI (axial T2WI and b 2000 s/mm^2^ DWI only) dataset were derived from mpMRI. Three experienced radiologists scored a-bpMRI, standard biparametric MRI (bpMRI) and mpMRI in separate sessions. Diagnostic accuracy and interreader agreement of a-bpMRI was tested for different positivity thresholds and compared to bpMRI and mpMRI. Predictive values of a-bpMRI were computed for lower levels of PCa prevalence to simulate a screening setting. The primary definition of clinically significant PCa (csPCa) was Gleason ≥ 4 + 3, or cancer core length ≥ 6 mm. (3) Results: The median age was 62 years, the median PSA was 6.8 ng/mL, and the csPCa prevalence was 40%. Using a cut off of MRI score ≥ 3, the sensitivity and specificity of a-bpMRI were 92% and 48%, respectively. There was no significant difference in sensitivity compared to bpMRI and mpMRI. Interreader agreement of a-bpMRI was moderate (AC1 0.58). For a low prevalence of csPCa (e.g., <10%), higher cut offs (MRI score ≥ 4) yield a more favourable balance between the predictive values and positivity rate of MRI. (4) Conclusion: Abbreviated bpMRI protocols could match the diagnostic accuracy of bpMRI and mpMRI for the detection of csPCa. If a-bpMRI is used in low-prevalence settings, higher cut-offs for MRI positivity should be prioritised.

## 1. Introduction

Multiparametric magnetic resonance imaging (mpMRI) plays a well-established role in the diagnostic workup of prostate cancer (PCa) [1]. The standard mpMRI protocol consists of multi-planar T2-weighted imaging (T2WI), diffusion-weighted imaging (DWI) and dynamic contrast-enhanced (DCE) sequences [2]. Given the constantly increasing demand for prostate MRI, the long scan times and high costs of mpMRI are limiting its widespread adoption in clinical practice; furthermore, concerns have been raised regarding the use of gadolinium-based contrast media [3]. As a consequence, shorter non-contrast protocols (i.e., bi-parametric MRI, bpMRI) may represent a potential solution to address these issues of mpMRI. Of note, evidence is accumulating that bpMRI may perform as well as mpMRI for PCa detection [4,5].

Moreover, interest has recently grown over prostate MRI as a tool for PCa screening [6,7,8,9,10]. In this scenario, where shorter scan lengths would have important health economic implications, abbreviated bpMRI (a-bpMRI) protocols could further improve MRI accessibility and reduce costs [11]. However, evidence on their diagnostic performance is limited, and potential limitations of abbreviated protocols (such as an increased number of indeterminate findings) should be considered. In addition, the optimal scoring approach for a-bpMRI protocols should be defined and tailored to the screening setting in terms of optimal cut-off scores and interpretation strategies (e.g., single versus double reading) [12].

To address these open questions, the aim of the present study is to investigate the diagnostic accuracy of a-bpMRI (<10 min scan) using template prostate mapping biopsies (TTPM) as the reference standard and to determine the best scoring approach to be used in a screening setting.

## 2. Materials & Methods

This study was conducted retrospectively on datasets acquired between 2012 and 2014 in the Prostate Imaging Compared to Transperineal Ultrasound-guided biopsy for significant prostate cancer Risk Evaluation (PICTURE) study [13], a paired-cohort confirmatory study designed to assess the diagnostic accuracy of mpMRI in men undergoing TTPM and targeted biopsy. Ethical approval for the original study was granted by London City Road and Hampstead National research ethics committee REC reference 11/LO/1657.

Inclusion and exclusion criteria, along with the complete study protocol, have been previously described [14]; briefly, treatment-naïve men were eligible for the study if they had undergone prior TRUS biopsy and were advised to undergo further biopsies as part of standard care.

Among the total participants to PICTURE (*n* = 249), 151 patients were involved in the present study. Patient selection was carried out blinded to clinical data and MRI findings, and with knowledge of the biopsy results, to obtain a predetermined prevalence of clinically significant cancer (i.e., 50% prevalence of Gleason 3 + 4 on TTPM biopsy), similar to that observed in another prospective, a TTPM-based cohort of men with clinical suspicion of PCa [15].

### 2.1. Imaging Protocols

All men underwent 3T mpMRI with a pelvic-phased array coil. The clinical mpMRI protocol consisted of the following sequences: axial and coronal T2WI, DWI including high b-value (b 2000 s/mm^2^) and apparent diffusion coefficient (ADC) map using multiple b-values (b 0, 150, 500, 1000 s/mm^2^), T1WI, and dynamic contrast enhancement with gadolinium (Magnevist). The total duration of the clinical mpMRI protocol was 28 min and 14 s (Table 1). Sequence details are reported in Supplementary Table S1.

The a-bpMRI dataset was generated separately from the original mpMRI dataset; a-bpMRI included axial T2WI and b 2000 s/mm^2^ DWI sequences only (estimated duration: 9 min and 13 s) (Table 1).

### 2.2. Image Analysis

All scans were reviewed by three dedicated radiologists (FG, MI, HS), all with more than 7 years of experience in prostate MRI reporting. Readers were blinded to clinical information, original mpMRI reports, and biopsy results. The review process was two-fold: first, a session for a-bpMRI reporting; then a second session for bpMRI and mpMRI reporting, with an interval of 5 months between the two sessions. No feedback was provided to the readers until the end of the study.

First session: abbreviated biparametric MRI

A-bpMRI scans were reported using a 5-point Likert scale for the likelihood of the presence of clinically significant cancer (1: highly unlikely; 2: unlikely; 3: equivocal; 4: likely; 5: highly likely) [16]. Likert scales are recommended for use in the UK by a consensus panel [17] and recent UK National Institute for Health and Care Excellence (NICE) guidance [18]; they were used for a-bpMRI scoring in the ReIMAGINE screening study [9]. The following information was also collected: image quality of T2WI and DWI sequences (i.e., clear delineation of anatomical structures, no major artefact preventing sequence interpretation), and Likert scores for individual T2WI/DWI sequences.

Second session: biparametric and multiparametric MRI

During the second session, readers first scored the bpMRI images, and then the full protocol (including DCE) was revealed for mpMRI scoring. BpMRI and mpMRI scans were reported using both Likert and PI-RADS v 2.1 score.

### 2.3. Histopathologic Assessment

All men underwent TTPM biopsies regardless of the MRI results within the PICTURE study [13]. Biopsies were performed using a brachytherapy grid fixed on a stepper, with cores taken every 5 mm. All biopsies were reported by one of two uropathologists with more than 20 years of experience, blinded to the mpMRI reports.

Clinically significant cancer was defined by criteria developed and validated for TTPM biopsies [19]. The primary definition (definition 1) of csPCa was considered the presence of dominant Gleason pattern 4 or greater (i.e., Gleason ≥ 4 + 3) or a cancer core length (CCL) involvement of ≥6 mm of any Gleason score. Alternative definitions were: (i) Gleason ≥ 3 + 4 and/or CCL ≥ 4 mm (definition 2), and (ii) presence of any Gleason ≥ 7.

### 2.4. Study Objectives

The primary objective of the study was to assess the diagnostic performance of a-bpMRI, alone and in comparison to bpMRI and mpMRI. The secondary objectives were: to assess interreader agreement of different MRI protocols, to investigate which lesions are missed by MRI using different protocols, to assess the performance of a-bpMRI for different cutoffs of MRI positivity, to simulate the performance of a-bpMRI in a low prevalence setting (e.g., screening), and to assess the performance of a double-reading approach.

### 2.5. Statistical Analyses

Contingency tables were used to calculate the diagnostic accuracy of the tests on a patient level for a cut off of MRI score ≥ 3: sensitivity, specificity, positive predictive value (PPV), negative predictive value (NPV), and MRI positivity rate (i.e., proportion of men with positive MRI finding according to cut-off score) were reported with 95% confidence intervals. True-positive, false-positive, true-negative, and false-negative results from each reader were combined by simple pooling to calculate diagnostic accuracy measures. Differences in sensitivity and specificity between a-bpMRI, bpMRI, and mpMRI were assessed using McNemar’s test, while differences in predictive values were assessed using a general estimating equation logistic regression model [20,21].

The diagnostic accuracy of a-bpMRI was then tested using alternative cut-off scores: (1) overall MRI score ≥ 4, and (2) the presence of any lesion with both T2WI and DWI scores ≥ 4. A-bpMRI scores from each reader were evaluated both individually and in combination (referred to as “combined scores”) to simulate a double-reading approach in which two radiologists report independently with a third reviewer involved when there is disagreement.

Finally, sensitivity and specificity values of a-bpMRI were used to predict the variation of PPV, NPV, and MRI positivity rates (i.e., the proportion of men with a positive MRI over the total of men undergoing MRI) for a lower prevalence of csPCa (2, 5, and 10%) [22].

Interobserver agreement was assessed using Gwet’s agreement coefficient 1 (AC1) and percentage of agreement (PA) [23,24]. Levels of agreement were interpreted using the classification of Landis and Koch [25].

Statistical tests were performed using RStudio graphical interface for R software v.4.0.2 (R Foundation, Vienna, Austria) and AgreeStat v.2015.6 (AgreeStat Analytics, Gaithersburg, MD, USA).

## 3. Results

### 3.1. Baseline Characteristics

A summary of the baseline demographic data of the men included in this study is presented in Table 2; the median age was 62 years (range: 41–83), and the median PSA was 6.8 ng/mL (range: 0.9–28.5). The prevalence of clinically significant cancer at TTPM biopsy was 60/151 (40%) according to definition 1, 95/151 (63%) according to definition 2, and 76/151 (50%) for any Gleason ≥ 7.

### 3.2. Diagnostic Accuracy of MRI

On a-bpMRI, axial T2WI sequences were judged to be of good diagnostic quality by all readers for all patients; DWI sequences were judged to be of low diagnostic quality in 10, 5, and 11 patients by the three readers, respectively, due to the presence of image artefacts.

The sensitivity, specificity, PPV, and NPV of a-bpMRI with a cut-off of MRI score ≥ 3 for the detection of definition 1 csPCa were 92% (95%—CI: 87–96), 48% (95%—CI 42–54), 54% (95%—CI: 48–60), and 90% (95%—CI: 84–95), respectively; there was no significant difference in sensitivity and NPV between a-bpMRI, bpMRI, and mpMRI (Table 3). The diagnostic accuracy of MRI for alternative definitions of csPCa is reported in Supplementary Table S2.

The proportions of MRI scores (1–2, 3, and 4–5) were similar for a-bpMRI, bpMRI, and mpMRI PI-RADS scores (32, 14, and 54% vs. 37, 13, and 50% vs. 36, 14, and 50%, respectively). A higher proportion of MRI score 3 was observed for bpMRI-mpMRI when using Likert scores (up to a 7% increase) (Supplementary Figure S1).

### 3.3. A-bpMRI: Alternative Cut-Offs and Combined Scores

Higher MRI cut-offs (MRI score ≥ 4 or higher) were associated with lower sensitivity (range: 70–83%) and higher specificity (range: 64–76%) for csPCa detection (Table 4; Supplementary Table S3); combined scores yielded slightly better performance, even if the difference was not significant.

### 3.4. Missed csPCa

Most of the lesions missed by mpMRI and a-bpMRI were of low-to-intermediate grade (Gleason 3 + 3 or 3 + 4; range *n* = 2–6), while one Gleason 4 + 3 tumour was missed by all MRI protocols regardless of the MRI cut-off (Supplementary Table S4). The number of missed high-grade tumours did not increase with a-bpMRI compared to mpMRI (*n* = 1). Conversely, it increased when a cut-off of MRI score ≥ 4, on both T2W and DWI, was used for a-bpMRI (*n* = 3, i.e., 2 additional Gleason 4 + 3 tumours).

### 3.5. Interreader Agreement

Interreader agreement of a-bpMRI was moderate and lower than mpMRI PI-RADS scores (PA: 76 vs. 81%; AC1 0.58 vs. 0.65, respectively); however, it was comparable to that of bpMRI (Supplementary Table S5). Agreement on a-bpMRI scores increased with higher positivity thresholds and was as high as 82% (CI—95%: 77–87; AC1 0.64, CI—95% 0.53–0.72) for a cut-off of MRI score ≥ 4 on both T2WI and DWI (Supplementary Table S6).

### 3.6. a-bpMRI and csPCa Prevalence

Assuming the sensitivity and specificity of MRI remained constant, predictive values were simulated for variable levels of csPCa prevalence (10, 5, and 2%). At lower levels of prevalence, PPV decreased while NPV increased; for a hypothetic csPCa prevalence of 5% as described in screening populations [26], NPV was very high (range: 98–100%) regardless the MRI cut-off used (Table 5; Supplementary Table S7). Higher MRI cut-offs were associated to lower positivity rates of MRI (Table 5).

## 4. Discussion

The present study provided evidence that an abbreviated biparametric MRI protocol consisting in axial T2WI and high b-value DWI sequences only (<10 min scan time) could match the performance of full biparametric and multiparametric MRI protocols for the detection of csPCa when interpreted by experienced radiologists. Our findings support emerging evidence on the limited utility of DCE for PCa detection [27,28], and are in line with similar studies that investigated abbreviated bpMRI protocols [11,29,30,31,32,33].

Fast MRI protocols that can be performed in less than 10 minutes could have a favorable impact on costs and accessibility of MRI [11]. As a consequence, bpMRI has recently drawn attention as a potential tool for imaging-based PCa screening, comparable to mammography for breast cancer or low-dose CT scan for lung cancer [6,7,8,9,10]. However, the screening setting is different from that of secondary care, where prostate MRI was developed, and little is known about the impact on MRI performance; notably, the prevalence of csPCa in the general population is significantly lower than in men with a clinical suspicion of PCa [26]. In this setting, attention should be focused on ruling out significant PCa while minimizing the number of positive MRI to avoid unnecessary interventions [3]. One of the most straightforward ways to achieve this goal is to use higher thresholds for MRI positivity. Accordingly, we simulated the performance of a-bpMRI at lower prevalence levels, and we observed that higher cut-offs (e.g., MRI score ≥ 4 or higher) yield a more acceptable balance between sensitivity and specificity, significantly reducing the proportion of positive scans and false-positive findings (Table 5). Concurrently, the NPV is expected to remain high (>97%) due to the low prevalence of the disease.

These numbers compare favorably with other established screening modalities; for lung cancer screening using low-dose CT, positivity rates of 27% and overall PPV of approximately 4% have been reported [34]. However, our findings should not be considered an exact prediction of the accuracy of MRI in a screening setting; instead, they are intended to provide an insight into the effect that different MRI cut-offs, scoring approaches, and disease prevalence have on the ability to predict the presence/absence of csPCa, in order to guide future applications of MRI for PCa screening. Real numbers are likely to be affected by true prevalence (which will vary with age and patient selection), prior tests, patient setting, scoring systems, biopsy techniques, and definitions of csPCa. In the recently published IP1-PROSTAGRAM study, where MRI was used as a primary screening test [8], a total of 17 clinically significant cancers were found in the 406 men screened (observed prevalence: 4.2%). Out of 406 men, 43 had a positive MRI using a cut-off of PI-RADS score 4–5 (scan positive rate: 10.6%); among these, csPCa was found in 11/43 men (PPV: 25.6%). Interestingly, these data overlap with those predicted in our study for a 5% prevalence of definition 2 csPCa (Supplementary Table S7).

One of the major drawbacks associated with the use of abbreviated MRI protocols is the potential increase in equivocal findings (i.e., PI-RADS/Likert score = 3) [11,35]. In our study, a-bpMRI did not lead to an increased number of equivocal findings compared to standard bpMRI and mpMRI, possibly due to the high experience of readers in prostate MRI interpretation. Moreover, the impact of equivocal scores would be negligible if a cut off of MRI score ≥ 4 is used [8]. Conversely, we observed lower interobserver agreement of a-bpMRI compared to previous reports [11,36]. One possible explanation is that T2WI, DWI, and DCE have an incremental value on lesion visibility, and the absence of multiplanar T2WI and ADC maps from our a-bpMRI protocol may have decreased the confidence of readers in determining MRI scores [37]. Furthermore, the use of Likert instead of PI-RADS for a-bpMRI could have played a role (Supplementary Table S5) [38].

In our study, we also simulated the performance of a double-reading approach (i.e., two radiologists report the MRI independently, with a third reviewer involved in case of disagreement), that replicates the methodology used for screening mammography [39]. We did not observe any significant benefit, or disadvantage, in terms of diagnostic performance of combined vs. individual reads (Table 4); however, this approach could still be considered as a potential solution to reduce the variability in MRI interpretation of abbreviated protocols; moreover, second reads could represent a future application of artificial intelligence tools [40].

A main strength of our study is that all patients underwent mapping biopsies regardless of the MRI results, increasing the reliability of sensitivity and specificity estimates compared to conventional targeted biopsy cohorts. Second, the multireader design averages the variable reader performance that can be encountered in the clinical routine, increasing the generalisability of the results. Third, a-bpMRI and bp/mpMRI were reported in two different sessions separated by a wash-out period. This may enhance the detection of small differences in the diagnostic accuracy between different study protocols when compared to standard sequential reading, wherein the results of one test will be invariably influenced by the other.

This study also has several limitations. First, patients were selected from a broader cohort of men included in a prospective trial [13], potentially introducing a selection bias. However, the demographics and csPCa prevalence were similar to those of other studies based on unselected populations [15], supporting the generalisability of our cohort.

Second, although we did not find significant differences in the performance of a-bpMRI, bpMRI, and mpMRI, the study was not specifically powered to detect such small differences, and our findings must be confirmed by larger studies. Third, Likert scores were used for a-bpMRI reporting, limiting the reproducibility of our results and the comparison with PI-RADS scores. However, although PI-RADS can be applied to bpMRI, this scoring system has not been conceived for short protocols (i.e.,: monoplanar, no ADC maps) [2]. Fourth, all the readers involved in the study are experienced readers from high volume centers, a factor that limits the applicability of our results to low-volume settings and less-experienced readers.

## 5. Conclusions

Abbreviated biparametric MRI (<10 min scan) can be as accurate as mpMRI for the detection of csPCa in the diagnostic setting when interpreted by expert readers. As far as the scan time, availability, cost, and acceptability are concerned, one might argue that an a-bpMRI could be the ideal tool for imaging-based screening programs for PCa. If used for this purpose, higher cut-offs (MRI score ≥ 4) could yield a more favorable balance between PCa detection and false-positive rates and could be preferred over lower cut-offs (MRI score ≥ 3). A double-reading approach might be considered to address the variability of MRI interpretation.

## Figures and Tables

**Table 1:** Protocol scan times (min:s).

Sequence	MpMRI	bpMRI	a-bpMRI
Localizer (T2WI—sagittal)	0:19	0:19	0:19
T2WI—axial	5:14	5:14	5:14
T2WI—coronal	5:55	5:55	-
DWI (b0, 150, 500, 1000 s/mm^2^)	5:17	5:17	-
DWI b 2000 s/mm^2^	3:40	3:40	3:40
T1WI	3:06	-	-
DCE	4:43	-	-
Total:	28:14	20:25	9:13

Scan time of mpMRI and estimated scan times of bpMRI and a-bpMRI protocols. T2WI: T2-weighted imaging; DWI: diffusion weighted imaging; T1WI: T1-weighted imaging; DCE: dynamic contrast enhanced imaging; mpMRI: multiparametric MRI; a-bpMRI: abbreviated biparametric MRI.

**Table 2:** Summary of demographic data.

No. of Patients	151
Median age, y (range)	62 (41–83)
Median PSA, ng/mL (range)	6.8 (0.9–28.5)
Highest Gleason grade at histopathology (%)	
Benign	22 (15)
3 + 3	53 (35)
3 + 4	63 (42)
≥4 + 3	13 (8)
Definition 1 * csPCa	60 (40)
Definition 2 ** csPCa	95 (63)
Any Gleason ≥ 3 + 4	76 (50)
Median no. of positive cores (IQR)	6 (2–11)
Median MCCL (IQR)	4 (1.5–7)

* Gleason ≥ 4 + 3 and/or cancer core length (CCL) involvement ≥ 6 mm. ** Gleason ≥ 3 + 4 and/or CCL ≥ 4 mm. csPCa: clinically significant prostate cancer; IQR: interquartile range; MCCL: maximum cancer core length.

**Table 3:** Diagnostic accuracy of MRI for definition 1 csPCa.

	a-bpMRI	bpMRI		mpMRI	
	Likert	Likert	PI-RADS	Likert	PI-RADS
Sensitivity	92 (87–96)	92 (87–96)	89 (83–93)	92 (87–96)	89 (83–93)
Specificity	48 (42–54) *	35 (29–41)	52 (46–58)	39 (33–45)	53 (47–59)
PPV	54 (48–60) *	48 (43–54)	56 (49–61)	50 (44–56)	56 (50–61)
NPV	90 (84–95)	87 (79–93)	88 (82–92)	88 (81–94)	88 (82–92)

Pooled values are reported as % (95%—CI). * *p* < 0.05 a-bpMRI vs bpMRI/mpMRI Likert. bpMRI: biparametric MRI; mpMRI: multiparametric MRI; a-bpMRI: abbreviated biparametric MRI; PPV: positive predictive value; NPV: negative predictive value.

**Table 4:** Pooled and combined diagnostic accuracy of a-bpMRI according to different MRI cut-offs (for definition 1 csPCa).

	MRI Score ≥ 4	T2WI and DWI Score ≥ 4
Pooled		
Sensitivity	83 (76–88)	70 (63–77)
Specificity	64 (58–69)	76 (71–81)
PPV	60 (54–66)	66 (59–73)
NPV	85 (79–89)	79 (74–84)
Combined *		
Sensitivity	85 (76–94)	72 (60–83)
Specificity	65 (55–75)	79 (71–87)
PPV	61 (51–72)	69 (58–81)
NPV	87 (79–95)	81 (73–89)

Pooled values (aggregate from the three readers) are reported as % (95%—CI). * Simulated double-reading approach: two radiologists report the a-bpMRI independently with a third reviewer involved when there is disagreement between the reporters. PPV: positive predictive value; NPV: negative predictive value.

**Table 5:** PPV, NPV, and positivity rates of abbreviated bpMRI (a-bpMRI) according to clinically significant PCa prevalence.

	Prevalence of csPCa (Definition 1)		
	10%	5%	2%
MRI score ≥ 3			
Pos.rate	59 (51–67)	57 (49–65)	56 (47–64)
PPV	16 (9–25)	8 (3–16)	4 (1–10)
NPV	98 (91–100)	100 (94–100)	100 (95–100)
MRI score ≥ 4			
Pos. rate	40 (32–49)	37 (30–46)	36 (29–45)
PPV	21 (12–34)	11 (4–22)	5 (1–15)
NPV	98 (92–100)	99 (94–100)	100 (96–100)
T2WI and DWI score ≥ 4			
Pos. rate	26 (19–34)	23 (17–31)	22 (16–29)
PPV	28 (15–45)	14 (5–30)	6 (1–20)
NPV	96 (91–99)	98 (94–100)	99 (95–100)

Values are reported as % (95%—CI). Pos. rate: rate of positive test according to MRI cut-off. csPCa: clinically significant prostate cancer; pos. rate: positivity rate of MRI; PPV: positive predictive value; NPV: negative predictive value.

## Data Availability

The data are not publicly available due to regulatory restrictions.

## Supplementary Materials

Supplementary Materials: The following are available online at https://www.mdpi.com/article/10.3390/diagnostics12020231/s1, Figure S1: Proportions of MRI scores, Table S1: MRI protocol details, Table S2: Diagnostic accuracy of MRI, Table S3: Diagnostic accuracy of a-bpMRI (combined scores), Table S4: Clinically significant lesions (definition 1) missed by MRI, Table S5: Interreader agreement (cut off: MRI score 3), Table S6: Interreader agreement of a-bpMRI using alternative MRI cut-offs, Table S7: NPV and positivity rates of abbreviated bp-MRI (a-bpMRI) according to clinically significant PCa prevalence.
