# Supplementary material for: Diagnostic Accuracy of Abbreviated Bi-Parametric MRI (a-bpMRI) for Prostate Cancer Detection and Screening: A Multi-Reader Study
Source: Diagnostics (Basel). 2022 Jan 18;12(2):231. doi: 10.3390/diagnostics12020231 (PMC8871361; doi:10.3390/diagnostics12020231)
Supplement: Supplementary file 1 [file diagnostics-12-00231-s001.zip › diagnostics-1511597-supplementary.pdf]

Supplementary material

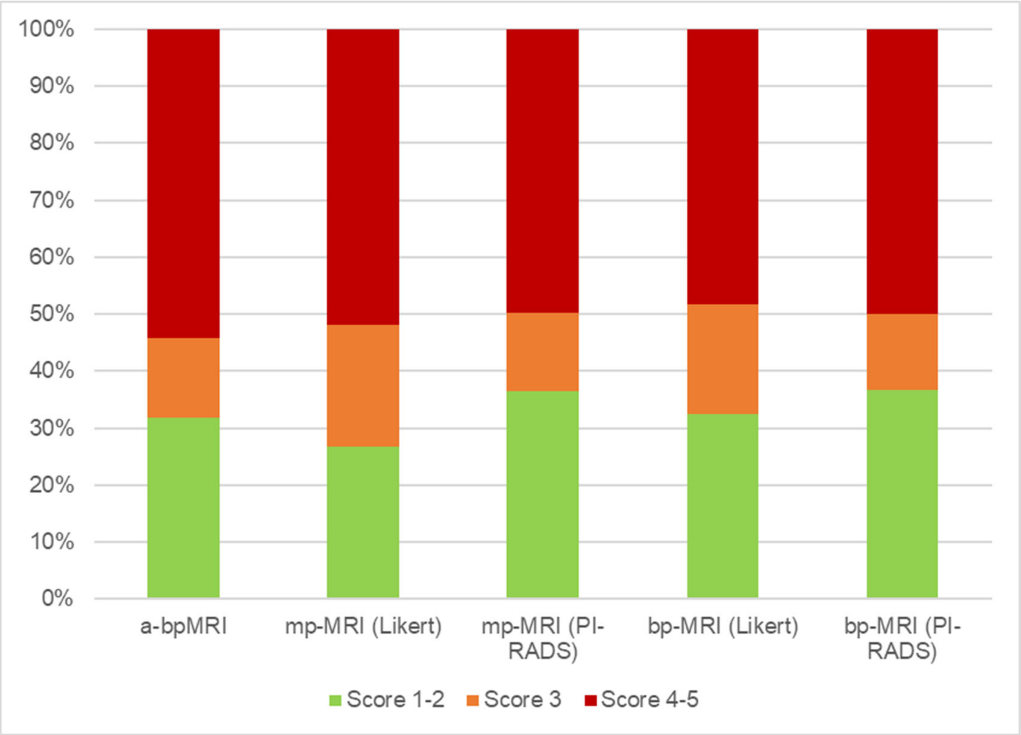

Figure S1. Proportions of MRI scores.MRI scores were grouped as follows: score 1-2, 3 and 4-5. MRI score distribution for a-bpMRI was 32%, 14% and 54%, respectively; for mp-MRI Likert scores was 27%, 21% and 52%, respectively; for mp-MRI PI-RADS scores was 36%, 14% and 50%; for bp-MRI Likert scores was 32%, 19% and 48%, respectively; for bp-MRI PI-RADS scores was 37%, 13% and 50%. Proportions are on the total number of patients included in the study (n = 151).

Table S1. MRI protocol details

Sequence	TR (ms)	TE (ms)	FoV (mm)	Slice thickness (mm)	Gap (mm)
T2 TSE coronal	6128	100	180	3	3
T2 TSE axial	5407	100	180	3	0
T1W TSE	487	8	240	3	3
DWI 0 150 500 1000	2753	80	220	5	0
DWI b2000	2000	78	220	5	0
DCE	5.8	2.8	180	3	0

Table S2. Diagnostic accuracy of MRI.

	a-bpMRI	bp-MRI (Likert)	bp-MRI (PI-RADS)	mp-MRI (Likert)	mp-MRI (PI-RADS)
Gleason ≥3+4 and/or MCCL ≥4 mm (definition 2); prevalence 63%					
Sensitivity	87 (83-91)	89 (85-93)	84 (79-88)	89 (85-93)	84 (79-88)
Specificity	65 (57-72)*	55 (48-63)	70 (62-76)	54 (46-62)	71 (64-78)
PPV	81 (76-85)	77 (72-82)	82 (78-87)	77 (72-81)	83 (79-87)
NPV	75 (67-82)	75 (66-82)	72 (65-79)	75 (67-83)	73 (65-79)
Any Gleason ≥3+4; prevalence 50%					
Sensitivity	88 (83-92)	90 (86-94)	86 (81-90)	91 (86-94)	86 (81-90)
Specificity	52 (45-59) * ‡	44 (39-52)	58 (51-64)	44 (38-51)	59 (52-66)
PPV	65 (59-70)	63 (57-68)	67 (62-73)	62 (57-68)	68 (62-73)
NPV	81 (73-87)	82 (74-89)	80 (73-86)	83 (75-89)	81 (74-86)

Pooled values are reported as % (95%-CI). *$p<0.05$ a-bpMRI vs bp/mp-MRI Likert. ‡ $p<0.05$ a-bpMRI vs bp/mp-MRI PI-RADS. bp-MRI: biparametric MRI; mp-MRI: multiparametric MRI; a-bpMRI: abbreviated biparametric MRI; PPV: positive predictive value; NPV: negative predictive value; MCCL: maximum cancer core length.

Table S3. Diagnostic accuracy of a-bpMRI (combined scores)

	**Definition 2 csPCa***	***Any Gleason ≥3+4***
	MRI score ≥4	
Sens	76 (67-84)	78 (68-87)
Spec	80 (70-91)	68 (57-79)
PPV	87 (79-94)	71 (61-81)
NPV	66 (55-77)	75 (65-85)
	T2 and DWI score ≥4	
Sens	60 (50-70)	62 (51-73)
Spec	91 (84-99)	80 (71-89)
PPV	91 (85-99)	76 (65-86)
NPV	57 (47-68)	67 (58-77)

Values are reported as % (95%-CI). *Gleason ≥3+4 and/or MCCL ≥4 mm. PPV: positive predictive value; NPV: negative predictive value

Table S4. Clinically significant lesions (definition 1) missed by MRI.

	Mp-MRI (Likert)	Mp-MRI (PI-RADS)	a-bpMRI
	Number (MCCL range, mm)		
**MRI score ≥3**			
Gleason 3+3	2 (7-10)	4 (6-10)	2 (6-10)
Gleason 3+4	1 (6)	2 (6-10)	0
Gleason 4+3	1 (4)	1 (4)	1 (4)
**MRI score ≥4**			
Gleason 3+3	4 (6-10)	6 (6-10)	5 (6-10)
Gleason 3+4	5 (6-10)	6 (6-10)	3 (6-10)
Gleason 4+3	1 (4)	2 (4-8)	1 (4)
**T2WI and DWI score ≥4**			
Gleason 3+3	na	na	7 (6-10)
Gleason 3+4	na	na	7 (6-12)
Gleason 4+3	na	na	3 (4-8)

The table refers to MRI lesions missed by at least 2 out of 3 readers using different cut-off for MRI positivity. MCCL: maximum cancer core length; na: not assessed.

Table S5. Interreader agreement (cut off: MRI score ≥3).

	Mp-MRI (Likert)	*Mp-MRI (PI-RADS)*	*Bp-MRI (Likert)*	*Bp-MRI (PI-RADS)*	*a-bpMRI*
Gwet's AC1	0.59 (0.48-0.69)	0.65 (0.56-0.75)	0.58 (0.48-0.69)	0.61 (0.51-0.71)	0.58 (0.48-0.69)
Percentage of agreement	75% (70-80)	81% (77-86)	75% (70-80)	79% (74-84)	76% (72-82)

Gwet's AC1: agreement coefficient which takes values between 0 to 1, similar to kappa.
Values in parentheses are 95%-CI.
mp-MRI: multiparametric MRI; a-bpMRI: abbreviated biparametric MRI.

Table S6. Interreader agreement of a-bpMRI using alternative MRI cut-offs

	MRI score ≥4	T2 ≥4 and DWI≥4
Gwet's AC1	0.59 (0.49-0.69)	0.64 (0.53-0.72)
Percentage of agreement	79% (74-84)	82% (77-87)

Gwet's AC1: agreement coefficient which takes values between 0 to 1, similar to kappa.
Values in parentheses are 95%-CI.

Table S7. NPV and positivity rates of abbreviated bp-MRI (a-bpMRI) according to clinically significant PCa prevalence.

	Prevalence of csPCa		
	**10%**	**5%**	**2%**
**Definition 2 csPCa***			
MRI score ≥3			
Pos. rate	42 (34-50)	38 (31-47)	37 (29-45)
PPV	22 (13-34)	12 (5-23)	5 (1-15)
NPV	99 (94-100)	99 (94-100)	100 (96-100)
MRI score ≥4			
Pos. rate	25 (18-33)	23 (16-30)	21 (14-28)
PPV	29 (15-46)	18 (7-35)	6 (1-21)
NPV	96 (91-99)	98 (94-100)	99 (95-100)
T2WI and DWI score ≥4			
Pos. rate	14 (9-20)	12 (7-18)	10 (6-16)
PPV	43 (22-66)	28 (10-53)	13 (2-40)
NPV	95 (90-98)	98 (94-100)	99 (96-100)
**Any Gleason ≥3+4**			
MRI score ≥3			
Pos. rate	52 (44-60)	50 (42-59)	49 (51-57)
PPV	18 (10-28)	9 (4-18)	4 (1-11)

NPV	99 (93-100)	100 (95-100)	100 (95-100)
MRI score ≥4			
Pos. rate	37 (29-45)	34 (27-42)	32 (25-41)
PPV	22 (12-35)	12 (4-23)	4 (0-14)
NPV	97 (91-99)	98 (93-100)	99 (95-100)
T2WI and DWI score ≥4			
Pos. rate	24 (17-31)	22 (16-30)	21 (15-29)
PPV	25 (12-42)	15 (5-31)	6 (1-21)
NPV	95 (89-98)	97 (93-99)	99 (95-100)

Values are reported as % (95%-CI). *Gleason ≥3+4 and/or MCCL ≥4 mm.

Pos. rate: rate of positive test according to MRI cut-off.

csPCa: clinically significant prostate cancer; pos. rate: positivity rate of MRI; PPV: positive predictive value; NPV: negative predictive value.
